# Evaluation of elite wheat (*Triticum aestivum* L.) genotypes for resistance to stem rust (*Puccinia graminis* f.sp. *tritici*), yield and yield stability

**DOI:** 10.3389/fpls.2025.1581007

**Published:** 2025-06-30

**Authors:** Jenniffer J. Kemboi, Sridhar Bhavani, Pascal P. Okwiri Ojwang

**Affiliations:** ^1^ Department of Crops, Horticulture and Soils, Egerton University, Egerton, Kenya; ^2^ Global Wheat Program-International Maize and Wheat Improvement Center (CIMMYT), International Centre for Research in Agroforestry (ICRAF) House, Nairobi, Kenya

**Keywords:** stem rust, bread wheat (*T. aestivum* L.), adult plant resistance, genotype by environment (G × E) interaction, wheat breeding for resistance

## Abstract

Stem rust, caused by *Puccinia graminis* f. sp. *tritici*, is a destructive fungal disease of bread wheat (*Triticum aestivum* L.) and poses a major challenge to wheat production in sub-Saharan Africa and Asia. The continuous evolution and variable nature of stem rust predispose wheat to serious genetic vulnerability, necessitating proactive incorporation of new and effective resistance sources into breeding lines. This study evaluated 25 wheat genotypes over three seasons at the Kenya Agricultural and Livestock Research Organization (KALRO), Njoro, to assess resistance mechanisms and yield stability under stem rust pressure. A 5 × 5 partially balanced alpha lattice design was employed. Disease progression was assessed using final disease severity (FDS) and area under the disease progress curve (AUDPC), alongside evaluations of agronomic performance. Statistical analyses revealed significant (*P* ≤ 0.001) effects of genotype, season, and genotype × season interaction for AUDPC and agronomic traits. Grain yield (GY) was significantly (*P* ≤ 0.001) negatively correlated with disease components, and positively correlated with kernels per spike (KS), biomass (BM), harvest index (HI), and thousand kernel weight (TKW). Broad-sense heritability (H^2^) estimates ranged from 59.90% for grain filling period to 95.58% for FDS. Adult plant resistance genes *Lr34/Yr18/Sr57*, *Lr46/Yr29/Sr58*, *Sr2/Yr30*, and *Lr67/Yr46/Sr55/Pm46* were detected in various combinations across 21 genotypes. Based on disease response and yield performance, genotypes 8790929, 8790027, 8790948, and 8790935 exhibited the highest levels of resistance and superior grain yield. These genotypes represent valuable sources of stem rust resistance and are recommended for use in breeding programs for gene introgression and varietal development.

## 1 Introduction

Stem rust (*Puccinia graminis* f.sp. *tritici*) is a devastating fungal disease threatening bread wheat (*Triticum aestivum* L.) and durum wheat (T. *turgidum* subsp. *durum*) production worldwide (Burrage, 1970; Bhavani et al., 2019). According to Leonard and Szabo (2005), a crop that appears healthy 3 weeks before harvest can be devastated by an explosive buildup of stem rust if sufficient inoculum arrives from a heavily infected wheat crop in some distant region. The infection of stem rust at extreme severities can result in a complete loss of a farmer’s crop (Newcomb et al., 2016). Stem rust has been reported to cause yield losses of up to 100% in susceptible varieties when the environmental conditions are favorable for the development of the disease (Leonard and Szabo, 2005). In recent years, stem rust has become significant due to occurrence of new virulent races which render commonly used varieties vulnerable (Figueroa et al., 2018; Singh et al., 2015). The ability of the rust pathogen to evolve novel, highly aggressive virulent races enables it to rapidly overcome host resistance conferred by the newly released resistant varieties (Roelfs et al., 1992).

Wheat stem rust has largely been controlled for over three decades due to the extensive use of resistant cultivars. Genetic resistance has been effectively used to protect wheat varieties against stem rust damage (Jin et al., 2007; Ellis et al., 2014). However, stem rust reemerged as a major threat in 1999 with the detection of a new race in Uganda with a notably unique virulence to widely deployed resistance gene *Sr31* (*Ug99*) (Pretorius et al., 2000), designated *TTKSK* according to the North American stem rust nomenclature system. After the identification of the *Ug99* strain *TTKSK*, 15 new variants of that lineage (*TTKSK, TTKSF, TTKST, TTTSK, TTKSP, PTKSK, PTKST, TTKSF+, TTKTT, TTKTK, TTHSK, PTKTK, TTHST, TTKTT+* and *TTHTT*) have appeared and overcome other resistance genes (International Maize and Wheat Improvement Center, Mexico, 2020). For instance, race *TTKST* with combined virulence on *Sr24* and *Sr31* detected in 2006 (Jin et al., 2008) resulted in severe localized epidemics in Kenya. The stem rust races of the *Ug99* (*TTKSK*) lineage carry complex virulence combinations and their migration to various countries in Africa, the Middle East and Asia continues to pose a significant threat to global wheat production (Bhavani et al., 2019). The emergence and spread of extremely virulent stem rust races and the high prevalence of the pathogen population in the key wheat-producing regions have rendered most commercial varieties susceptible to stem rust (Figueroa et al., 2018). The rapid movement of stem rust races of *Ug99* and non-*Ug99* lineage from eastern Africa to other regions (Singh et al., 2006) has made breeding for cultivars with combinations of effective resistance genes important globally to mitigate the threat of this devastating disease (Jin et al., 2008; Singh et al., 2015).

More effective sources of resistance need to be identified and incorporated in the existing commercial cultivars (Njau et al., 2010). The two main classes of genes that are useful to develop resistance are race specific (R) genes and race non-specific genes or adult plant resistance (APR) genes. Race specific genes are effective from seedling to adult plant growth stage while race non-specific (APR) genes are mainly effective at adult growth stage (Huerta-Espino et al., 2011). Over the years, seedling and adult plant resistance genes for stem rust have been studied and catalogued in wheat and its wild relatives (Ellis et al., 2014; Aktar-Uz-Zaman et al., 2017). To date, 66 stem rust *(Sr)*, 80 Yellow rust *(Yr)* and 100 Leaf rust (*Lr)* resistance genes have been identified and catalogued (Kumar et al., 2022; McIntosh et al., 2020). However, the majority of these resistance genes have been identified to confer race-specific resistance (Mapuranga et al., 2022). Therefore, more focus is to develop wheat cultivars with slow rusting resistance genes that confer durable resistance to wheat stem rust (Mabrouk et al., 2019). Slow rusting type of resistance is both race non-specific and durable and is characterized by a slow disease build up despite a high infection indicating a compatible host-pathogen relationship (Priyamvada et al., 2011; Mabrouk et al., 2019). The slow rusting genes have been the backbone of resistance in the Mexican germplasm since 1950s (Huerta-Espino et al., 2020). This type of resistance is polygenic and effective against a broad range of rust races and can be measured in the field by recording disease severity at weekly intervals and then calculating the area under the disease progress curve (AUDPC) (Wilcoxson et al., 1975; Herrera-Foessel et al., 2007). The effects of these APR genes when alone, are moderate but they play an important role in gene combinations and interactions with other R (major) genes and a range of other minor QTLs that cause additive effects, resulting in high levels of durable resistance (Huerta-Espino et al., 2020; Singh et al., 2015). Concerted research efforts have resulted in the identification of several new resistance genes and gene combinations for use in breeding. In recent years, progress has been made in combining multiple APR genes in high-yielding backgrounds and discovery of new QTLs conferring stem rust resistance, thus aiding in enhancing the durability of resistance (Bhavani et al., 2019; Singh et al., 2011a).

Quantitative traits such as adult plant resistance and yield are attributed to inherent performance of a genotype, the influence of the genotype by environmental conditions in which a genotype grows and the genotype × environment interaction (GEI) (Kang, 1997; Yan and Hunt, 2001). Measurement of GEI enables the plant breeder to identify consistently well ranked genotypes that display stable performance across locations within the target region over a number of seasons and/or years, including disease conditions (Yan and Tinker, 2005). Understanding the effect of environment on phenotypic expression of wheat genotypes on a number of important traits and the sensitivity of wheat genotypes to environmental changes is important in identifying high yielding and stable wheat genotypes with improved adaptation to changing environments and have a key role in assessment of stability of the breeding materials (Mohammadı et al., 2012; Gupta et al., 2022). Given that grain yield trait is controlled by additive effect genes and is highly influenced by the environment, it becomes the most important priority trait for selection in all breeding programs (Braun et al., 2010). Yield stability has always been considered as an important phenomenon in plant breeding especially due to the continued variation in environmental conditions. Thus, successful phenotypic evaluation of elite breeding lines leads to identification of genotypes that display high and stable grain yield performance across varying environments for target traits (Yan and Tinker, 2005). The Mexico-Kenya shuttle breeding scheme that maintains two crop seasons per year in both Mexico and Kenya has also enhanced the development of wheat germplasm with broad adaptation to diverse wheat growing environments. Furthermore, this breeding strategy has resulted in the identification of rare transgressive segregants that combine high yield potential with stem rust resistance (Lantican et al., 2016; Bhavani et al., 2019). The objective of this study is to determine the adult plant resistance to stem rust race *Ug99* in the elite wheat lines with high grain yield potential.

## 2 Materials and methods

### 2.1 Experimental site

The experiment was conducted at the International Stem Rust (*Ug99*) Phenotyping Platform at the Kenya Agricultural and Livestock Research Organization (KALRO), Food Crops Research Institute, Njoro. The phenotypic platform (0˚ 20’S, 32˚ 56’ E) is located at an elevation of 2,293 meters above sea level. The location receives approximately 1,000 mm of precipitation per annum with mean annual minimum and mean annual maximum temperatures of 8°C and 25°C, respectively. The location is situated within the lower highland III (LH_3_) agro-ecological zone and has well-drained Mollic andosol soils (Jaetzold et al., 2012). The site was selected because it favors prevalence of the epidemics of stem rust and yellow rust on wheat and barley (*Hordeum vulgare*).

### 2.2 Genotypes for the study

A set of twenty-five (25) wheat lines from CIMMYT nurseries pre-selected for stem rust resistance was used in this study (**Table 1**). These genotypes were developed from crosses of diverse parents followed by different selection histories in the KALRO-CIMMYT shuttle program. Molecular analysis of the 21 elite wheat lines was previously performed using validated molecular markers and the postulated genes are presented in **Table 1**. These wheat lines developed by CIMMYT are available for identification and release as wheat varieties in Kenya. The checks used were selected from genotypes that are resistant, moderately resistant and susceptible against the stem rust.

**Table 1:** Pedigree crosses and postulated genes of CIMMYT elite wheat germplasm used in the experiment.

Ent	GID	Cross/Pedigree information	Postulated genes
1	8789894	MERCATO/VORB*2/4/SWSR22T.B.//TACUPETOF2001*2/BRAMBLING/3/2*TACUPETO F2001*2/BRAMBLING	Lr46 [Syn.=Yr29=Sr58=Pm39=Ts?=Ltn2]+Sr22+Se2
2	8790025	KIRITATI//HUW234+LR34/PRINIA/3/CHONTE/5/PRL/2*PASTOR/4/CHOIX/STAR/3/HE1/3*CNO79//2*SERI*2/6/BORL14	Lr46 [Syn.=Yr29=Sr58=Pm39=Ts?=Ltn2]+Lr34 [Syn. =Yr18=Sr57=Pm38=Sb1=Bdv1=Fhb?=Ltn1] +SrHuw234+Sr2?
3	8790026	KIRITATI//HUW234+LR34/PRINIA/3/CHONTE/5/PRL/2*PASTOR/4/CHOIX/STAR/3/HE1/3*CNO79//2*SERI*2/6/BORL14	Lr46 [Syn.=Yr29=Sr58=Pm39=Ts?=Ltn2]+Lr34 [Syn. =Yr18=Sr57=Pm38=Sb1=Bdv1=Fhb?=Ltn1] +SrHuw234+Sr2?
4	8790027	KIRITATI//HUW234+LR34/PRINIA/3/CHONTE/5/PRL/2*PASTOR/4/CHOIX/STAR/3/HE1/3*CNO79//2*SERI*2/6/BORL14	Lr46 [Syn.=Yr29=Sr58=Pm39=Ts?=Ltn2]+Lr34 [Syn. =Yr18=Sr57=Pm38=Sb1=Bdv1=Fhb?=Ltn1] +SrHuw234+Sr2?
5	8790046	KIRITATI//HUW234+LR34/PRINIA/3/CHONTE/5/PRL/2*PASTOR/4/CHOIX/STAR/3/HE1/3*CNO79//2*SERI*2/6/KINGBIRD #1//INQALAB 91*2/TUKURU	Lr46 [Syn.=Yr29=Sr58=Pm39=Ts?=Ltn2]+Lr34 [Syn. =Yr18=Sr57=Pm38=Sb1=Bdv1=Fhb?=Ltn1] +SrHuw234+Lr67+Sr2?
6	8790048	KIRITATI//HUW234+LR34/PRINIA/3/CHONTE/5/PRL/2*PASTOR/4/CHOIX/STAR/3/HE1/3*CNO79//2*SERI*2/6/KINGBIRD #1//INQALAB 91*2/TUKURU	Lr46 [Syn.=Yr29=Sr58=Pm39=Ts?=Ltn2]+Lr34 [Syn. =Yr18=Sr57=Pm38=Sb1=Bdv1=Fhb?=Ltn1] +SrHuw234+Lr67+Sr2?
7	8790075	PBW343*2/KUKUNA//PBW343*2/KUKUNA/3/WBLL1*2/SHAMA//KACHU/4/KASUKO/5/KASUKO	Lr46 [Syn.=Yr29=Sr58=Pm39=Ts?=Ltn2]+Sr2+Sr8115b+Sr2?
8	8790258	KENYA TAI*2/5/FRANCOLIN #1/3/PBW343*2/KUKUNA*2//YANAC/4/KINGBIRD #1//INQALAB 91*2/TUKURU	SrND643+SrYanac+ Lr46 [Syn.=Yr29=Sr58=Pm39=Ts?=Ltn2]+Sr2?
9	8790275	KENYA SUNBIRD/4/KACHU*2/3/ND643//2*PRL/2*PASTOR/5/SWSR22T.B./2*BLOUK #1//WBLL1*2/KURUKU	SrND643+SrYanac+ Lr46 [Syn.=Yr29=Sr58=Pm39=Ts?=Ltn2]+Sr2?
10	8790311	KASUKO/4/CIRO16*2/3/MUU #1/SAUAL//MUU/5/KASUKO	Lr46 [Syn.=Yr29=Sr58=Pm39=Ts?=Ltn2]+Sr8115b+Sr2?
11	8790384	BAJ #1*2/PREMIO/4/BOKOTA*2/3/UP2338*2/KKTS*2//YANAC/9/KFA/2*KACHU*2/8/TACUPETO F2001/6/CNDO/R143//ENTE/MEXI_2/3/AEGILOPSSQUARROSA(TAUS)/4/WEAVER/5/PASTOR/7/ROLF07	Lr46 [Syn.=Yr29=Sr58=Pm39=Ts?=Ltn2]+SrYananc+SrSha7+Sr2?
12	8790668	WARRIOR,GBR/CIRO16/9/TRAP#1/BOW/3/VEE/PJN//2*TUI/4/BAV92/RAYON/5/KACHU #1/8/2*TACUPETOF2001/6/CNDO/R143//ENTE/MEXI_2/3/AEGILOPSSQUARROSA(TAUS)/4/WEAVER/5/PASTOR/7/ROLF07	Lr46 [Syn.=Yr29=Sr58=Pm39=Ts?=Ltn2]+SrShah7+Sr2?
13	8790751	SR50/4/3*KACHU*2/3/ND643//2*PRL/2*PASTOR	Lr46 [Syn.=Yr29=Sr58=Pm39=Ts?=Ltn2]+Sr50+SrND643+Sr2?
14	8790754	SR50/4/3*KACHU*2/3/ND643//2*PRL/2*PASTOR	Lr46 [Syn.=Yr29=Sr58=Pm39=Ts?=Ltn2]+Sr50+SrND643+Sr2?
15	8790800	SR50/4/3*KACHU*2/3/ND643//2*PRL/2*PASTOR	Lr46 [Syn.=Yr29=Sr58=Pm39=Ts?=Ltn2]+Sr50+SrND643+Sr2?
16	8790806	SR50/4/3*KACHU*2/3/ND643//2*PRL/2*PASTOR	Lr46 [Syn.=Yr29=Sr58=Pm39=Ts?=Ltn2]+Sr50+SrND643+Sr2?
17	8790929	SR50/4/3*KACHU*2/3/ND643//2*PRL/2*PASTOR	Lr46 [Syn.=Yr29=Sr58=Pm39=Ts?=Ltn2]+Sr50+SrND643+Sr2?
18	8790935	SR50/4/3*KACHU*2/3/ND643//2*PRL/2*PASTOR	Lr46 [Syn.=Yr29=Sr58=Pm39=Ts?=Ltn2]+Sr50+SrND643+Sr2?
19	8790948	SR50/4/3*KACHU*2/3/ND643//2*PRL/2*PASTOR	Lr46 [Syn.=Yr29=Sr58=Pm39=Ts?=Ltn2]+Sr50+SrND643+Sr2?
20	8790874	W3763-SR35/4/3*KACHU*2/3/ND643//2*PRL/2*PASTOR	Lr46 [Syn.=Yr29=Sr58=Pm39=Ts?=Ltn2]+Sr35+SrND643+Sr2?
21	8790885	W3763-SR35/4/3*KACHU*2/3/ND643//2*PRL/2*PASTOR	Lr46 [Syn.=Yr29=Sr58=Pm39=Ts?=Ltn2]+Sr35+SrND643+Sr2?
22	-	PBW 343	Check
23	-	KENYA ROBIN	Check
24	-	KINGBIRD	Check
25	-	KENYA KASUKU	Check

Source: CIMMYT. GID, genotype identification number.

### 2.3 Experimental procedure

Twenty-five (25) wheat genotypes were evaluated over three seasons [off-season (OS) 2022, main-season (MS) 2022 and off-season (OS) 2023] at the International Stem Rust Phenotyping Platform at, KALRO, Njoro. The seasons hereafter are designated as OS-2022, MS-2022 and OS-2023. The land previously under canola (*Brassica napus*) crop was ploughed and harrowed to a fine tilth using a disc plough and a harrow, respectively. A rotavator was used to turn the soil until the seed bed was fine and levelled. The seeds of each genotype were sown in furrows of double rows measuring 70 × 20 cm at the seed rate of 125 kg ha^-1^ and Di-ammonium phosphate (DAP 18:46:0) fertilizer was applied in furrows before sowing at the rate of 130 kg ha^-1^ to supply 23 kg N ha^-1^ and 60 kg P_2_O_5_ ha^-1^ (Kamwaga et al., 2016). The experiment was set up in a 5×5 partially balanced *square lattice design* with 3 replications and each replicate was separated by 50 cm alley. The experimental trial consisted of 5 blocks per replicate with each block containing 5 genotypes.

A continuous double row of a spreader mixture of susceptible wheat lines was planted surrounding the experimental plot to build uniform disease pressure. Spreader mixture was also planted as hill plots in a way that each row was surrounded by spreader mixture on one side. Artificial stem rust epidemic was created by inoculating the spreader rows commencing at stem elongation stage (GS 30-39) (Zadoks et al., 1974) with bulk inoculum of fresh rust spores collected from disease nursery. Inoculation was done 2 times a week until the disease developed on the spreader rows. For this experiment, inoculation was done by injection using a syringe and spraying using a hand sprayer and using two rust suspensions, one with Tween 20^R^ solution and the other with Soltrol 170^R^ Isoparaffin solvent, respectively. Two to three plants randomly selected per meter were inoculated with a syringe at the peduncle with stem rust inoculum. Overhead irrigation was set up each day for 3 hours in the evening for the days that did not receive rainfall on the day of inoculation to enhance epiphytotic conditions.

At tillering stage (GS 20-29), top dressing with urea (46:0:0) was done at the rate of 100 kg ha^-1^ to supply 46 kg N ha^-1^. Wide-ranging grasses and broad-leaved weeds were controlled by using a pre-emergence herbicide, Stomp^®^ 455 CS (*Pendimethalin* 455 g/L). A post-emergence herbicide, Huskie^®^ 256 EC (*Pyrasulfotole* 37.5 g/L + *Bromoxynil* 210 g/L + *Safener Mefenpyr-diethyl* 9.38 g/L) was also used to control broadleaved weeds. A systemic insecticide, Thunder^®^ OD 145 (B*eta-cyfluthrin* 0.009 kg ha^-1^ + *Imidacloprid* 0.02 kg ha^-1^) at 0.029 kg active ingredient ha^-1^ was applied to control Russian wheat aphids (*Diuraphis noxia*) as soon as the first signs of infestations were observed during tillering and booting (GS 40-49) stage.

### 2.4 Data collection

Stem rust severity was evaluated as percent leaf and stem area infected for each plot starting at heading stage (GS 50 to 59) to ripening stage (GS90 to GS99) over 3 consecutive weeks with an interval of 7 days. The disease severity was estimated based on modified Cobb’s scale (Peterson et al., 1948) with a severity rating of 0 to 100%, where 0 = immune and 100 = highly susceptible depending on the extent of the area affected by the disease. The host plant response (HPR) to stem rust infection was determined according to the size of pustules and associated necrosis and/or chlorosis. The HPRs were designated as Resistant (R) = small uredinia surrounded by necrosis, Resistant to Moderately Resistant (RMR), Moderately Resistant (MR) = small to medium uredinia surrounded by chlorosis or necrosis, Moderately Resistant to Moderately Susceptible (M) = small to medium uredinia surrounded by chlorosis and medium-sized uredinia that may be associated with chlorosis, Moderately Susceptible (MS) = medium-sized uredinia that may be associated with chlorosis, Moderately Susceptible to Susceptible (MSS) = medium to large uredinia with very few or no chlorosis and Susceptible (S) = large uredinia without chlorosis or necrosis (Roelfs et al., 1992).

The Area under Disease Progress Curve (AUDPC) was calculated using the disease severity estimates following the formula below (Wilcoxson et al., 1975).


$$\text{AUDPC}=\sum_{i=1}^{n}(\frac{\left(y_{i}+y_{i+1}\right)}{2}(t_{i+1}-t_{i}))$$

where $y_{i}$ is the disease observation (severity) on the $i^{th}$ scoring; $t_{i}$ is the number of each reading in days from sowing to $i^{th}$ scoring; *n* is the total number of scores; $t_{i+1}$ is second assessment date of two consecutive assessments and is disease severity on the assessment date (i+1. The resistance of genotypes was compared using AUDPC and Final Disease Severity (FDS) data which was the average disease severity during the final score across the three seasons.

Data for yield and other yield-related traits were collected on plant and plot basis. On plant basis, the data collected included plant height (cm), spike length and the number of kernels per spike. The days to heading, days to flowering, total biomass, harvest index, 1000-kernel weight (g) and grain yield data were collected on plot basis. Plant height was measured as the mean vertical distance from the ground level to the tip of the spikes from 5 randomly selected wheat plants in a plot. The mean spike length was determined as a measure from the first spikelet node to the spike tip, excluding the awns, whereas the mean number of kernels spike^-1^ was determined as the number of seeds from 5 randomly selected spikes.

Days to heading was obtained when at least 50% of the test genotypes’ spikes had emerged from the boot from sowing, while days to flowering was determined when 50% of the plants had flowered in a given plot. At physiological maturity, plots were harvested by cutting at the base and total biomass was estimated using an electronic weighing balance. The grain yield was determined by measuring the weight of the kernels in a given plot using an electronic weighing scale after sun drying when the moisture content was 12%. The grain filling period was then computed by determining the time the photosynthates took to fill the kernels from anthesis to maturity. The harvest index (HI) was also estimated from the ratio of the total grain yield to the total biomass of plants in a given plot. 1000-kernel weight (TKW) was determined by counting 1,000 kernels on a Contador seed counter (serial number 14176107) and weighing them using a weighing balance.

### 2.5 Data analyses

Prior to analyses, AUDPC data was log transformed to obtain a normal frequency distribution and back transformed to the original scale for presentation in tables and figures. The data were then subjected to restricted maximum likelihood (REML) analysis (Patterson and Thompson, 1971) to obtain the variance components, using GenStat 15^th^ edition computer software programme (VSN International, Hemel Hempstead, UK). The linear mixed model (LMM) was used as given below:


$$y_{ijkl}=\mu +R_{k(j)}+G_{i}+S_{j}+GS_{ij}+\beta_{l(kj)}+{\text{ϵ}}_{ijkl}$$

where $y_{ijkl}$ is the response, µ is the overall mean, $R_{k(j)}$ is the effect due to the k^th^ replicate nested within the j^th^ season, $G_{i}$ is the effect due to the i^th^ genotype, $S_{j}$ is the effect due to j^th^ season, $GS_{ij}$ is the effect due to the interaction between the i^th^ genotype and the j^th^ season, $\beta_{l(kj)}$ is the effect due to l^th^ block nested within k^th^ replicate and j^th^ season and ${\text{ϵ}}_{ijkl}$ is the random error component. The effects due to replicates, genotypes and seasons were considered as fixed whereas the effect due to incomplete blocks nested within replicates considered as random. The least significant difference (LSD) test was adopted for mean separation.

Correlation analysis was conducted in GenStat to establish the relationship between stem rust (AUDPC), final disease severity (FDS), grain and related traits. Given *n*, the correlation coefficient (r) between any two variables was determined as follows;


$$r=\frac{n\sum x_{i}y_{i}-(\sum x_{i})(\sum y_{i})}{\sqrt{\left[n\sum x_{i}^{2}-{\left(\sum x_{i}\right)}^{2}].[n\sum y_{i}^{2}-{\left(\sum y_{i}\right)}^{2}\right]}}$$

where *r* is Pearson’s correlation coefficient, n is the number of observations, $(x_{i},\;y_{i}),i=$ 1 , …, n is the number pairs of observations.

The phenotypic coefficient of variation (PCV) and genotypic coefficient of variation (GCV) were determined as follows;


$$PCV\;(\%)=\;\frac{\sqrt{\sigma_{P\;\;}^{2}}\;\;}{\mu }\;\times \;100\%\text{ and }GCV\;(\%)=\;\frac{\sqrt{\sigma_{g}^{2}}}{\mu }\;\times \;100\%$$

where $\sigma_{P\;\;}^{2}$ is the variance due to phenotype and $\sigma_{g}^{2}$ is the genotypic variance and μ is the grand mean.

Broad sense heritability (H^2^) for each trait over seasons were estimated regarding genotypes and season as random effects and replicates as fixed as shown in the equation given below. Heritability percentages of 0–30%, 30–60% and ≥ 60% were classified as low, moderate and high, respectively (Johnson et al., 1955).


$$H^{2\;\;}=\;\frac{\sigma_{g}^{2}}{\sigma_{g}^{2}+(\frac{\sigma_{gs}^{2}}{s}+\;\frac{\sigma_{e}^{2}}{sr})}$$

where $\sigma_{g}^{2}$ is the genotypic variance, $\sigma_{gs}^{2}$ is the genotype-by-season interaction variance, *s* is the number of seasons, $\sigma_{e}^{2}$ is the residual variance and *r* is the number of replications.

The joint regression analysis (JRA) was used to assess genotype adaptation across seasons (Finlay and Wilkinson, 1963) following the model;


$$y_{ij}=\;\text{μ}+\;G_{i}+\;{\text{ β}}_{i}E_{j}+R_{k(j)}+\beta_{l(kj)}+\;\epsilon_{ij}$$

where $G_{i}$ is the genotypic main effect, ${\text{ β}}_{i}$ is the slope reaction norm, $E_{j}$ is the seasonal main effect, $R_{k(j)}$ is the replicate effect, $\beta_{l(kj)}$ is the block effect nested within replicate and season and $\epsilon_{ij}$ is the random error component. The JRA aims at assessing how the expected performance of a genotype varies as a function of the environmental effects.

## 3 Results

### 3.1 Variance components

Residual maximum likelihood analyses revealed significant main effects (P ≤ 0.001) of genotype and season for all traits (**Appendix 1**). Significant (P ≤ 0.001) genotype-by-season interaction (GSI) effects were found for area under disease progress curve (AUDPC), days to heading (DH), days to flowering (DF), days to physiological maturity (DM), grain filling period (GFP), number of kernels per spike (KS^-1^), grain yield (GY) and a 1000-kernel weight (TKW). Significant (P ≤ 0.01) effects due to genotype-by-season interaction was also found on plant height (PH) and harvest index (HI) and significant (P ≤ 0.05) for spike length (SL) and final disease severity (FDS). However, GSI was not significant for biomass (BM).

### 3.2 Genotypic mean performance

**Figure 1:**
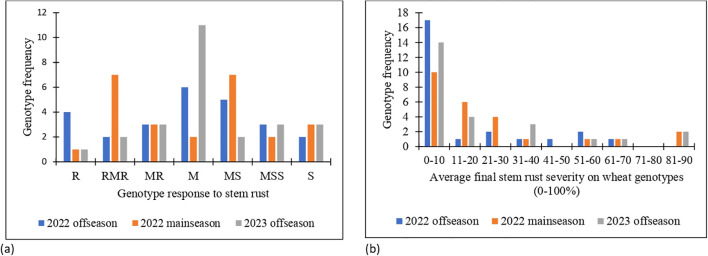
**(a)** Response of the wheat genotypes to stem rust and **(b)** frequency distribution of average final stem rust severity in 2022 off-season, 2022 main-season and 2023 off-season.

The means for area under disease progress curve (AUDPC) and final disease severity (FDS) were 157, 227 and 210 and 15.5, 24.3 and 21.9 during the 2022 off-season, 2022 main-season and 2023 off-season, respectively (**Appendix 2**). The AUDPC and FDS mean values observed were lower in 2022 off-season compared to 2022 main-season and 2023 off-season. This trend showed that the stem rust disease pressure was high during the 2022 main-season and is explained by high disease severity and AUDPC values among the genotypes. AUDPC values of the genotypes ranged from 2 to 793, 32 to 968 and 18 to 1015 and FDS ranged from 1 to 70, 5 to 87 and 5 to 90 in 2022 off-season, 2022 main-season and 2023 off-season, respectively (**Appendix 2**). More than half of the wheat genotypes exhibited average final disease severity range of 0 to 20% with resistant (R) to MS host response over the seasons. The frequency distribution of the genotypes for wheat response formed a normal distribution and skewed to the left for stem rust severity (**Figures 1a, b**).

All 21 CIMMYT genotypes screened carried the adult plant resistance genes *Lr46/Yr29/Sr58* and *Sr2/Yr30*. Additionally, three genotypes also possessed *Lr34/Yr18/Sr57*, and two genotypes contained all four APR genes (**Table 2**). CIMMYT genotypes with genotype identification numbers (GID) 8789894 (G1), 8790027 (G4), 8790046 (G5), 8790751 (G13), 8790800 (G15), 8790929 (G17), 8790935(G18), 8790948 (G19) and 8790874 (G20) displayed resistance to stem rust across the three seasons. The host plant response (HPR) displayed by these genotypes ranged from resistant (R) to moderately resistant (MR). On the other hand, the HPR of the susceptible checks (PBW 343, Kenya Robin and Kenya Kasuku) ranged from moderately susceptible to susceptible (MSS) to susceptible (S) and moderately resistant and moderately susceptible (M) to moderately susceptible (MS) (**Appendix 2**).

**Table 2:** List of markers linked to seedling and adult plant resistance genes with their respective primer sequence.

Marker name	Marker type	Allele-specific primer	
Sr35	KASP	**A1**	TGCTTTTGTCTCGGTTTCGCA
		**A2**	TGCTTTTGTCTCGGTTTCGCG
		**C**	TCGTCTATGAGATACTTTTCGTCCC
Sr25_CAP7_c2912_1387634	KASP	**A1**	AACCGGTTACAAAGCCAAATCCAGA
		**A2**	CCGGTTACAAAGCCAAATCCAGG
		**C**	ACTAGTGCTTGGTTTACCAATGTTCCTA
Sr38	KASP	**A1**	GGACGGCGTTTGCTCATGCTA
		**A2**	AGGACGGCGTTTGCTCATGCTG
		**C**	AGCAGTATGTACACAAAA
Sr23_kwm847	KASP	**A1**	GTAACCACGGTGAAGCTGGCG
		**A2**	GTAACCACGGTGAAGCTGGCA
		**C**	TTGTTGTGCCGCCAGCCTCCAT
Sr23_kwm849	KASP	**A1**	TGGCTTCGCGATGTCCACA
		**A2**	GGCTTCGCGATGTCCACG
		**C**	AGCGCCAAGTAGGCCATGCAGA
Sr26_sunKASP_224	KASP	**A1**	GAGCAGATGAGGAAAAGAGGC
		**A2**	GAGCAGATGAGGAAAAGAGGA
		**C**	CTTCCGCCCTGTGTATTTCG
Sr26_sunKASP_225	KASP	**A1**	CCAAGAATCACACACCATAGGTG
		**A2**	CCAAGAATCACACACCATAGGAT
		**C**	CCCTACAACTGCACCGATGT
Sr58_SNP1G22*	KASP	**A1**	ACCCATGGCTTTGGCTCCG
		**A2**	CTACCCATGGCTTTGGCTCCA
		**C**	GAAATACGCTAAGACGCCTCCATCAT
Sr57_csLV34	STS	**F**	GTTGGTTAAGACTGGTGATGG
		**R**	TGCTTGCTATTGCTGAATAGT
ND643-gwm350	SSR	**F**	ACCTCATCCACATGTTCTACG
		**R**	GCATGGATAGGACGCCC
Sr50-5p	STS	**F3**	TTCAGTGAAGTTGCCGCTGT
		**R2**	GCATGCTCTCAAGCTCCTTCT
Sr22-CFA2019	STS	**F**	GACGAGCTAACTGCAGACCC
		**R**	CTCAATCCTGATGCGGAGAT
Sr24#50	STS	**F**	CCCAGCATCGGTGAAAGAA
		**R**	ATGCGGAGCCTTCACATTTT

A1 - primer labelled with FAM, A2 - primer labelled with HEX, C - common primer, F – forward primer sequence, R - reverse primer sequence, * - personal communication, ** - FAM fluorescence = GAAGGTGACCAAGTTCATGCT and HEX fluorescence = GAAGGTCGGAGTCAACGGATT.

Days to heading (DH) had the same mean value of 69 cm across seasons indicating that it was not significantly affected by seasonal variation (**Table 3**). Days to physiological maturity (DM), grain filling period (GFP), plant height (PH), biomass (BM) and grain yield (GY) mean values were higher in 2023 off-season as compared with 2022 off-season and 2022 main-season (**Table 3**). This means that genotypes took longer to reach physiological maturity and had longer grain-filling period resulting in higher grain yields in the 2023 off-season. On the other hand, days to flowering (DF) and 1000-kernel weight (TKW) means were higher in 2022 main-season with 76 days and 31.26 g compared to 73 days and 28.90 g in 2022 off-season and 75 days and 28.29 g in 2023 off-season, respectively. Spike length (SL) was not significantly different in 2022 main-season and 2023 off-season with a mean of 9 cm (**Table 3**).

**Table 3:** The effect of the seasons on the means of agronomic traits of wheat lines evaluated across 3 cropping seasons at KALRO, Njoro.

Season	Days to				SL cm	PH (cm)	KS^-1^	BM (t ha^-1^)	GY (t ha^-1^)	HI	TKW (g)
	DH	DF	DM	GFP							
22OS	69	73	116	43	10	91	54	19.34	4.52	0.22	28.9
22MS	69	76	124	47	9	92	49	23.82	3.07	0.13	31.26
23OS	69	75	128	52	9	96	50	30.51	5.36	0.18	28.29
Mean	69	75	123	47	9	93	51	24.56	4.32	0.18	29.48
LSD_0.05_	0.54	0.42	0.53	0.61	0.14	1.20	1.81	1.61	0.30	0.01	0.56

22OS, 2022 off-season; 22MS, 2022 main-season; 23OS, 2023 off-season; DH, days to heading; DF, days to flowering; DM, days to physiological maturity; GFP, grain filling period; SL, spike length; PH, plant height; KS^-1^, kernels per spike; BM, biomass; GY, grain yield; HI, harvest index; TKW, 1000-kernel weight.

Mean grain yield (GY) was high in 2023 off-season with 5.36 t ha^-1^ compared to 4.52 t ha^-1^ in 2022 off-season and 3.07 t ha^-1^ in 2022 main-season (**Table 3**). This can be explained by the long DM and GFP observed during the same period. Genotypes 8789894 (G1), 8790258 (G8), 8790929 (G17), 8790935 (G18) and 8790948 (G19) had the highest overall grain yield means with 6.37, 6.70, 6.89, 6.61 and 7.14 t ha^-1^, respectively (**Appendix 3**). These genotypes yielded significantly higher than the check K. Kingbird with the overall mean of 3.53 t ha^-1^ across seasons. Harvest index (HI) and kernels per spike (KS^-1^) were higher in 2022 off-season and 2023 off-season than in 2022 main-season (**Table 3**). Genotype 8790027 (G4) emerged as the best-performing genotype in terms of HI with an average of 0.23 followed by 8790258 (G8) and 8790929 (G17) with the same HI of 0.22 across the three seasons (**Appendix 3**). On the other hand, in terms of KS^-1^, 16 genotypes including two checks (Kenya Kingbird and Kenya Kasuku) had an average of more than 50 kernels per spike across the three seasons while the check PBW 343 with 38.4 had the lowest mean value of kernels per spike (**Appendix 3**). Four genotypes namely 8789894 (G1), 8790929 (G17), 8790935 (G18) and 8790948 (G19) were observed to have combined stem rust resistance and high grain yields across seasons.

### 3.3 Correlation analysis among agronomic traits and stem rust severity

The area under disease progress curve (AUDPC) was highly correlated with final disease severity (FDS) (0.997***) (**Table 4**). The AUDPC was negatively correlated with days to heading (DH) (-0.424*), biomass (BM) (-0.598***), grain filling period (GFP) (-0.455*), grain yield (GY) (-0.750***), harvest index (HI) (-0.847***) and 1000-kernel weight (TKW) (-0.837***) (**Table 4**). The negative correlations between AUDPC and maturity indicate that the genotypes matured early due to high disease pressure while at the same time the disease pressure negatively affected productivity. FDS was negatively correlated with DH (-0.421*), BM (-0.577**), GFP (-0.480*), GY (-0.742***), HI (-0.845***) and TKW (-0.831***). The relationship between FDS and GY and other yield-related traits was comparable to AUDPC which elucidated disease impact on agronomic traits.

**Table 4:** Correlation of area under disease progress curve, final disease severity score and agronomic traits of elite wheat lines.

Traits	AUDPC	FDS	Days to heading	Days to maturity	Biomass	GFP	Grain yield	Harvest index	Kernels spike^-1^	Plant height
FDS	0.997***									
Days to heading	0.028	0.046								
Days to maturity	-0.424*	-0.421*	0.702***							
Biomass	-0.598**	-0.577**	0.129	0.448*						
GFP	-0.455*	-0.480*	-0.540**	0.190	0.253					
Grain yield	-0.750***	-0.742***	-0.173	0.201	0.880***	0.375				
Harvest index	-0.847***	-0.845***	-0.325	0.005	0.523**	0.369	0.833***			
Kernels spike^-1^	-0.353	-0.311	0.061	0.135	0.537**	-0.012	0.519**	0.432*		
Plant height	0.068	0.083	0.568**	0.423*	0.424*	-0.353	0.190	-0.115	0.309	
1000-kernel weight	-0.837***	-0.831***	-0.172	0.217	0.799***	0.394	0.922***	0.843***	0.464*	0.1792

AUDPC, area under disease progress curve; FDS, final disease severity and GFP, grain filling period. *, ** and *** = significant at p ≤ 0.05, p ≤ 0.01 and p ≤ 0.001, respectively.

The relationship between yield and yield-related traits varied. DH was positively correlated with DM (0.702***) and PH (0.568**) and negatively correlated with GFP (-0.540**). DM was positively associated with BM (0.448*) and PH (0.423*) (**Table 4**). Furthermore, BM was correlated with GY (0.880***), HI (0.523**), kernels spike^-1^ (KS^-1^) (0.537**), PH (0.424*) and TKW (0.799***). GY was positively correlated with HI (0.833***), KS^-1^ (0.519**) and TKW (0.922***) and KS^-1^ was positively correlated with TKW (0.464*).

### 3.4 Heritability, genotypic stability and adaptability analyses

Genotypic variance exceeded variance due to genotype-by-season interaction (GSI), variance due to season and variance due to error for AUDPC, FDS, days to heading (DH), days to flowering (DF), biomass (BM), grain yield (GY) and 1000-kernel weight (TKW) (**Table 5**). On the other hand, variance due to season exceeded variance due to genotype, variance due to GSI and error for days to maturity (DM) and grain filling period (GFP) but variance due to error was higher than seasonal variance, genotypic variance and variance due to GSI for plant height (PH), spike length (SL) and kernels per spike (KS^-1^). Harvest index (HI) had the lowest variance values for season, genotype, GSI and error while BM had the highest phenotypic variance. Phenotypic coefficient of variation (PCV) was higher than genotypic coefficient of variation (GCV) for all the traits. PCV ranged from 0.33% for AUDPC to 46.35% for GY. On the other hand, GCV ranged from 0.29% for AUDPC to 37.69% for GY.

**Table 5:** Estimates of variation and heritability for disease and agronomic traits of wheat lines evaluated over three cropping seasons.

Trait	σ^2s^	σ^2g^	σ^2g.s^	σ^2e^	σ^2p^	PVC (%)	GVC (%)	H^2^ (%)
AUDPC	0.059	0.322	0.024	0.073	0.418	0.33	0.29	95.25
FDS	0.041	0.186	0.013	0.037	0.237	2.37	2.10	95.58
Days to heading	0.166	13.182	1.986	2.831	17.999	6.16	5.27	93.10
Days for flowering	2.112	12.137	1.482	1.721	15.340	5.23	4.65	94.66
Days to maturity	33.588	6.527	7.690	2.676	16.893	3.36	2.09	69.53
Grain filling period (days)	21.924	4.019	6.886	3.552	14.457	8.00	4.22	59.90
Plant height (cm)	6.920	8.020	4.060	13.760	25.840	5.48	3.05	73.56
Spike length (cm)	0.069	0.174	0.031	0.205	0.410	6.92	4.51	84.10
Kernels spike^-1^	6.430	11.590	10.850	31.780	54.220	14.53	6.72	61.85
Biomass (t ha^-1^)	31.150	39.540	3.180	25.210	67.930	33.56	25.60	91.10
Grain yield (t ha^-1^)	1.314	2.643	0.505	0.851	3.999	46.35	37.69	90.96
Harvest index	0.002	0.001	0.001	0.002	0.004	35.30	20.38	75.28
1000-kernel weight (g)	2.157	33.168	6.354	3.018	42.540	22.12	19.54	93.11

σ^2s^, seasonal variance; σ^2g^, genotypic variance; σ^2g.s^, variance due to genotype by season interaction, σ^2e^, variance due to error; σ^2p^, phenotypic variance; PCV, phenotypic coefficient of variation; GCV, genotypic coefficient of variation; H^2^, heritability in broad-sense; AUDPC, area under disease progress curve and FDS – final disease severity.

Moderate to high broad-sense heritability (H^2^) estimates were recorded. H^2^ estimates ranged from 59.90% for the grain filling period to 95.58% for the final disease severity. All the traits were highly heritable, except for the grain filling period which had moderate heritability (**Table 5**).

**Figure 2:**
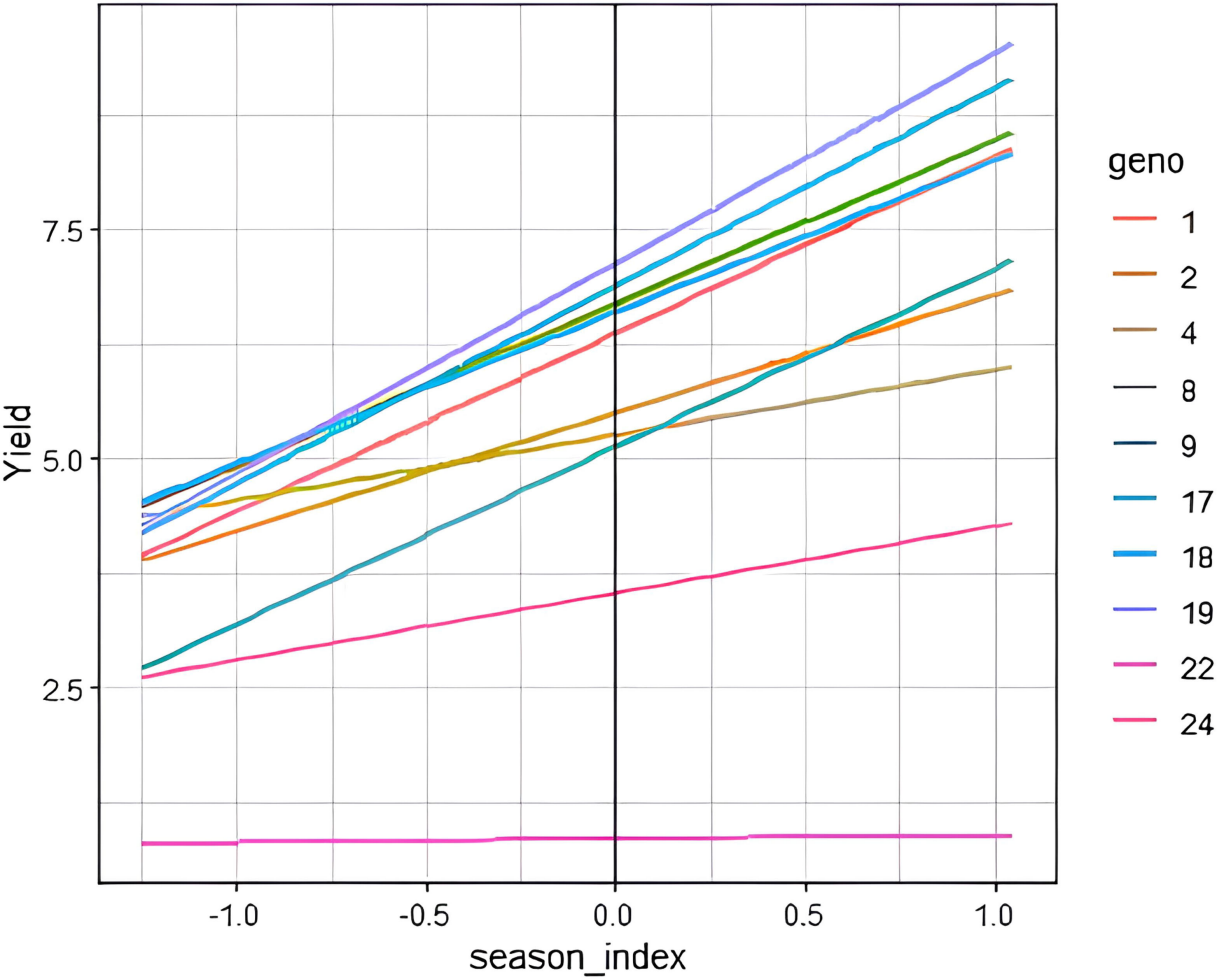
Performance of selected genotypes on estimated seasonal indices. Yield (t ha^-1^). Each colored line represents fitted values for means of genotype by season interaction: Checks: 22 – PBW 343 and 24 – Kingbird.

**Figure 3:**
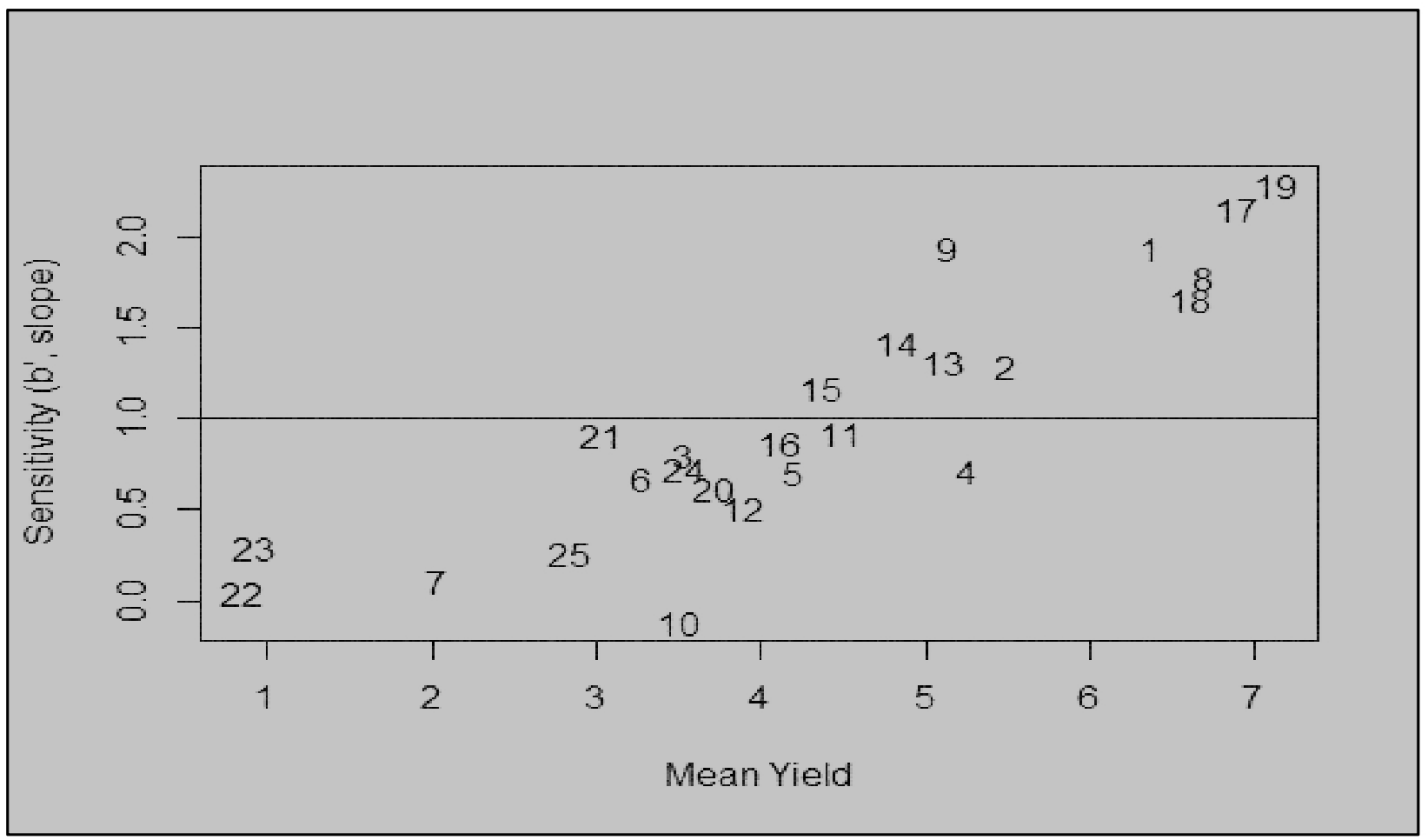
Sensitivity (b’) versus the mean yield (t ha^-1^); the relationship of genotype adaptation and genotypic mean yield for the genotypes representing specific adaptability to favourable environments, general adaptability, and specific adaptability for unfavourable environments across the seasons.

The modified joint regression analysis for yield revealed consistent performance of genotypes 8790948 (G19) and 8790929 (G17) across seasons (**Figure 2**). Apart from above average yield stability, these genotypes were resistant to stem rust (**Appendix 2**). In contrast, a local check PBW 343 (G22) consistently ranked low across seasons. The two-dimensional scatter plot displays genotypic adaptability to multiple seasons (**Figure 3**). From the scatter plot, genotype 8790311 (G10) located towards the bottom of the plot was associated with unfavourable cropping season. On the other hand, genotypes located towards the top of the plot comprising 8790275 (G9), 8789894 (G1), 8790929 (G17) and 8790948 (G19) were specifically adapted to favourable cropping seasons. Generally, the genotypes that are generally adapted to all seasons are located on or close to b=1.0 regression coefficient line. The further a genotype is to the right, the higher the yield.

## 4 Discussion

Stem rust is one of the most destructive wheat diseases and can cause heavy yield loss if not controlled (Singh et al., 2011b) especially with the constant evolution of new aggressive races. Improving wheat for resistance to stem rust is a significant step towards reducing damage caused by this disease in key production regions particularly in eastern Africa. Residual maximum likelihood (REML) analyses revealed significant genotype by season interaction (GSI) for area under disease progress curve (AUDPC) and final disease severity (FDS) across seasons. AUDPC and FDS have been used as reliable measures of adult plant resistance and therefore good indicators of wheat genotypes with slow rusting genes (Wilcoxson et al., 1975; Herrera-Foessel et al., 2007; Netsanet, 2017). Genotypes with low AUDPC values i.e. < 100, low FDS values i.e. < 30 were considered to have high levels of adult plant/slow rusting resistance in the study. Therefore, selection of lines with lower AUDPC values is acceptable for practical purposes. This was in agreement with previous studies that used AUDPC and FDS to assess slow rusting behavior of wheat lines (Huerta-Espino et al., 2020; Singh et al., 2008; Wasike et al., 2022).

Breeding genotypes for stem rust resistance is a continuous process and new effective sources of resistance need to be deployed in breeding programs. Genotypes 8790027 (G4), 8790929 (G17), 8790935 (G18) and 8790948 (G19) showed resistant (R) to resistant to moderately resistant (RMR) responses across the seasons. These genotypes possess a combination of adult plant and race-specific resistance genes that confer resistance to stem rust at the adult plant stage. The pathogen race specific genes are effective at all plant growth stages whereas adult plant resistance genes (APR) are usually functional only in adult plant stage. In contrast to most R genes, the levels of resistance conferred by single APR genes are only partial and allow considerable disease development (Ellis et al., 2014). Durable resistance which is controlled by minor genes and is long-lasting when deployed can be achieved by accumulating 4–5 minor genes into the same genetic background and these minor genes include *Lr34/Yr18/Sr57, Lr46/Yr29/Sr58, Sr2/Yr30* and *Lr67/Yr46/Sr55/Pm46* (Huerta-Espino et al., 2020; Singh et al., 2005). Notably, these four pleotropic genes that confer multi-pathogen resistance were identified in CIMMYT wheat genotypes screened. It has also been suggested that high levels of rust resistance can also be achieved when a moderately effective race-specific gene is combined with APR genes (Basnet et al., 2015, 2014; Singh et al., 2008). Inheritance of resistance of durable resistance indicates that the genotypes often carry a few slow rusting genes that have small-to-intermediate, but additive effects (Bhavani et al., 2022; Singh et al., 2005). CIMMYT- derived semi-dwarf wheat cultivar, Kingbird, used as a check in this study is known to carry *Sr2* gene and showed adequate levels of APR to *Ug99* race group. Genotypes with this gene show maximum disease severity of 10% - 15% with moderately resistant to moderately susceptible (M) to moderately susceptible (MS) reactions (Bhavani et al., 2019; Khan et al., 2013).

Effects due to genotypes were significantly different for all the traits indicating presence of high level of genetic diversity among genotypes. There was significant variation in genotypic performance among the three seasons. This could be attributed to diverse genetic backgrounds of the genotypes, weather conditions of seasons, disease pressure in that particular season and GSI effects. The trend showed a general reduction in performance for grain yield (GY) and related traits among the genotypes with an increase in stem rust infections. For instance, GY and kernels per spike (KS^-1^) reduced from 4.52 t ha^-1^ to 3.07 t ha^-1^ and 54 to 49 kernels with an increase in AUDPC and FDS from 157 to 227 and 15.5 to 24.3 in 2022 off-season and 2022 main-season, respectively. Previous studies showed that the high disease scores observed led to low yield (Cheruiyot et al., 2015; Macharia and Wanyera, 2012; Ogutu, 2017). Significant genotype-by-season interaction was observed for all the traits under study except for biomass (BM). This indicated differential response of genotypes to environments resulting in non-uniform phenotypic response of lines to stem rust, GY and yield-related traits. This variation could be either due to interaction of the genetic and non-genetic factors during plant growth as observed in other studies (Akçura et al., 2005; Msundi et al., 2021).

The AUDPC and FDS was negatively correlated to GY and yield components, this revealed that an increase in AUDPC and FDS led to a reduction in GY, thousand kernel weight (TKW), harvest index (HI), kernels per spike (KS^-1^), days to maturity (DM), grain filling period (GFP) and biomass (BM). This relationship indicates that there is a reduction in grain yield with an increase in stem rust infections. The effects of stem rust on GY in wheat evaluated across different environments in Kenya have been previously reported (Macharia and Wanyera, 2012). Stem rust affects the photosynthetic ability of the plant and the photosynthates transport system from the green part of the plant to the affected parts resulting in shrivelled grains and reduction in grain size (Leonard and Szabo, 2005; Rehman et al., 2013) The temporary accumulation of photosynthates in the stems of wheat near the time of anthesis contributes to an increase in kernel dry weight. However, if SR infection occurs during this critical period, it is likely to negatively impact GY (Romig and Calpouzos, 1970).

Grain yield was highly and positively correlated with TKW (0.922) and BM (0.880) indicating that TKW and BM have direct effect on yield and therefore can be used as a criterion for selecting superior genotypes. Therefore, simultaneous selections for the above traits for individual genotype will improve both traits. A study to evaluate wheat for yield and its components also revealed a highly positive relationship between GY and BM (Geleta et al., 2015). Phenotypic coefficient of variation (PCV) and genotypic coefficient of variation (GCV) estimates indicated the presence of a significant amount of variability among the genotypes for all the studied traits. In this study, all the traits had higher PCV compared to GCV, but the difference was very minimal signifying less influence of the environment. All the traits under study had high heritability except grain filling period indicating the predominance of additive gene effect and hence selection based on phenotypic performance for these traits would be effective (Dey et al., 2019).

According to Finlay and Wilkinson (1963) regression coefficients, genotypes characterized by b=1.0 are considered to have average phenotypic stability hence adapted to all environments, those with b>1 are highly adapted to high-yielding environments while those with b<1 are adapted to poor environment. The inbreds that had b<1 such as 8790311 (G10) are specifically adapted to less productive environments. Inbreds characterized by b=1.0 such as 8790384 (G11) and 8970885 (G21) expressed average phenotypic stability across seasons. Inbreds with b>1, for example, 8789894 (G1), 8790929 (G17) and 8970948 (G19) showed sensitivity to environmental changes and therefore highly adapted to favourable conditions. Selection for specific and broad adaptability is useful because farmers can utilize high-yielders cultivars for their respective environments. Similar results have also been reported (Mevlüt et al., 2009).

## 5 Conclusion

The assessment of the response of genotypes to stem rust and their yield performance across seasons revealed the presence of adult plant resistance to *UG99* races. Significant genotype-by-environment interaction was observed for grain yield. Specifically, wheat lines coded 8790027, 8970929, 8790935and 8790948 were superior for disease resistance and had high and stable grain yield across seasons. These genotypes have been identified to possess a combination of APR and major genes in their genetic background. This highlights the importance of employing molecular breeding in conjunction with phenotypic breeding in identifying the gene combinations in genetic material and their effect in slowing disease development. The identified genotypes are promising candidates for future varietal development targeting stem rust resistance in wheat and hold significant potential as parental lines in breeding programs aimed at enhancing rust resistance.

## Data Availability

The original contributions presented in the study are included in the article/supplementary material. Further inquiries can be directed to the corresponding authors.
