# Supplementary material for: Evaluation of elite wheat (Triticum aestivum L.) genotypes for resistance to stem rust (Puccinia graminis f.sp. tritici), yield and yield stability
Source: Front Plant Sci. 2025 Jun 30;16:1581007. doi: 10.3389/fpls.2025.1581007 (PMC12256480; doi:10.3389/fpls.2025.1581007)
Supplement: Supplementary file 1 [file Table1.docx]

**Supplementary material**

**APPENDIX 1** Combined REML variance component analyses for selected parameters of 25 wheat genotypes evaluated for stem rust resistance over three cropping seasons in 2022 and 2023 at KALRO, Njoro.

i.**Response variate: Area under disease progress curve**

Fixed model: Constant + replicate + genotype + season + genotype.season

Random model: Replicate.block

Number of units: 225

**Estimated variance components**

Random term component s.e.

Replicate.block -0.00341 0.00176

**Residual variance model**

Term Model(order) Parameter Estimate s.e.

Residual Identity Sigma2 0.0730 0.00890

**Deviance: -2*Log-Likelihood**

Deviance d.f.

-157.17 146

**Tests for fixed effects**

Fixed term Wald statistic n.d.f. F statistic d.d.f. F pr

Replicate 7.32 2 3.66 1.8 0.235

Genotype 1445.10 24 61.00 8.7 <0.001

Season 124.11 2 62.05 134.8 <0.001

Genotype.season 94.89 48 1.98 134.8 0.001

**Dropping individual terms from full fixed model**

Fixed term Wald statistic n.d.f. F statistic d.d.f. F pr

Replicate 7.32 2 3.66 1.8 0.235

Genotype.season 94.89 48 1.98 134.8 0.001

**Standard errors of differences**

Replicate Season Genotype Genotype.season

Average 0.04413 0.04413 0.1134 0.2131

Maximum 0.1215 0.2207

Minimum 0.1080 0.2101

**ii. Response variate: Final disease severity**

Fixed model: Constant + replicate + genotype + season + genotype.season

Random model: Replicate.block

Number of units: 225

**Estimated variance components**

Random term component s.e.

Replicate.block -0.00212 0.00062

**Residual variance model**

Term Model(order) Parameter Estimate s.e.

Residual Identity Sigma2 0.0371 0.00446

**Deviance: -2*Log-Likelihood**

Deviance d.f.

-260.42 146

**Tests for fixed effects**

Fixed term Wald statistic n.d.f. F statistic d.d.f. F pr

Replicate 18.50 2 9.25 1.0 0.224

Genotype 2390.05 24 985.58 2.1 <0.001

Season 168.39 2 84.20 138.2 <0.001

Genotype.season 100.14 48 2.09 138.2 <0.001

**Dropping individual terms from full fixed model**

Fixed term Wald statistic n.d.f. F statistic d.d.f. F pr

Replicate 18.50 2 9.25 1.0 0.224

Genotype.season 100.14 48 2.09 138.2 <0.001

**Standard errors of differences**

Replicate Season Genotype Genotype.season

Average 0.01181 0.1573 0.1488 0.2338

Maximum 0.1534 0.2724

Minimum 0.1446 0.2224

**iii. Response variate: Days to heading (days)**

Fixed model: Constant + replicate + genotype + season + genotype.season

Random model: Replicate.block

Number of units: 225

**Estimated variance components**

Random term component s.e.

Replicate.block 0.062 0.146

**Residual variance model**

Term Model(order) Parameter Estimate s.e.

Residual Identity Sigma2 2.827 0.343

**Deviance: -2*Log-Likelihood**

Deviance d.f.

394.12 146

**Tests for fixed effects**

Fixed term Wald statistic n.d.f. F statistic d.d.f. F pr

Replicate 0.04 2 0.02 6.2 0.978

Genotype 1021.87 24 42.26 110.8 <0.001

Season 15.04 2 7.52 136.1 <0.001

Genotype.season 149.25 48 3.11 136.1 <0.001

**Dropping individual terms from full fixed model**

Fixed term Wald statistic n.d.f. F statistic d.d.f. F pr

Replicate 0.04 2 0.02 6.2 0.978

Genotype.season 149.25 48 3.11 136.1 <0.001

**Standard errors of differences**

Replicate Season Genotype Genotype.season

Average 0.2746 0.2746 0.8106 1.383

Maximum 0.8146 1.386

Minimum 0.7997 1.373

**iv. Response variate: Days to flowering (days)**

Fixed model: Constant + replicate + genotype + season + genotype.season

Random model: Replicate.block

Number of units: 225

**Estimated variance components**

Random term component s.e.

Replicate.block 0.113 0.121

**Residual variance model**

Term Model(order) Parameter Estimate s.e.

Residual Identity Sigma2 1.721 0.209

**Deviance: -2*Log-Likelihood**

Deviance d.f.

324.27 146

**Tests for fixed effects**

Fixed term Wald statistic n.d.f. F statistic d.d.f. F pr

Replicate 3.12 2 1.56 7.3 0.273

Genotype 1426.13 24 59.20 130.6 <0.001

Season 191.24 2 95.62 135.8 <0.001

Genotype.season 171.97 48 3.58 135.8 <0.001

**Dropping individual terms from full fixed model**

Fixed term Wald statistic n.d.f. F statistic d.d.f. F pr

Replicate 3.12 2 1.56 7.3 0.273

Genotype.season 171.97 48 3.58 135.8 <0.001

**Standard errors of differences**

Replicate Season Genotype Genotype.season

Average 0.2142 0.2142 0.6496 1.089

Maximum 0.6571 1.094

Minimum 0.6306 1.071

**v. Response variate: Days to physiological maturity (days)**

Fixed model: Constant + replicate + genotype + season + genotype.season

Random model: Replicate.block

Number of units: 225

**Estimated variance components**

Random term component s.e.

Replicate.block 0.092 0.146

**Residual variance model**

Term Model(order) Parameter Estimate s.e.

Residual Identity Sigma2 2.647 0.320

**Deviance: -2*Log-Likelihood**

Deviance d.f.

385.57 146

**Tests for fixed effects**

Fixed term Wald statistic n.d.f. F statistic d.d.f. F pr

Replicate 5.73 2 2.86 7.1 0.123

Genotype 727.01 24 30.12 120.1 <0.001

Season 1922.64 2 961.32 136.7 <0.001

Genotype.season 466.84 48 9.73 136.7 <0.001

**Dropping individual terms from full fixed model**

Fixed term Wald statistic n.d.f. F statistic d.d.f. F pr

Replicate 5.73 2 2.86 7.1 0.123

Genotype.season 466.84 48 9.73 136.7 <0.001

**Standard errors of differences**

Replicate Season Genotype Genotype.season

Average 0.2657 0.2657 0.7920 1.343

Maximum 0.7977 1.346

Minimum 0.7768 1.328

**vi. Response variate: Grain filling period (days)**

Fixed model: Constant + replicate + genotype + season + genotype.season

Random model: Replicate.block

Number of units: 225

**Estimated variance components**

Random term component s.e.

Replicate.block 0.382 0.305

**Residual variance model**

Term Model(order) Parameter Estimate s.e.

Residual Identity Sigma2 3.526 0.427

**Deviance: -2*Log-Likelihood**

Deviance d.f.

433.14 146

**Tests for fixed effects**

Fixed term Wald statistic n.d.f. F statistic d.d.f. F pr

Replicate 0.91 2 0.46 8.3 0.649

Genotype 361.15 24 15.02 138.4 <0.001

Season 946.55 2 473.28 136.1 <0.001

Genotype.season 329.62 48 6.87 136.1 <0.001

**Dropping individual terms from full fixed model**

Fixed term Wald statistic n.d.f. F statistic d.d.f. F pr

Replicate 0.91 2 0.46 8.3 0.649

Genotype.season 329.62 48 6.87 136.1 <0.001

**Standard errors of differences**

Replicate Season Genotype Genotype.season

Average 0.3066 0.3066 0.9442 1.567

Maximum 0.9594 1.577

Minimum 0.9081 1.533

**vii. Response variate: Spike length (cm)**

Fixed model: Constant + replicate + genotype + season + genotype.season

Random model: Replicate.block

Number of units: 225

**Estimated variance components**

Random term component s.e.

Replicate.block -0.0089 0.0046

**Residual variance model**

Term Model(order) Parameter Estimate s.e.

Residual Identity Sigma2 0.198 0.0240

**Deviance: -2*Log-Likelihood**

Deviance d.f.

-8.72 146

**Tests for fixed effects**

Fixed term Wald statistic n.d.f. F statistic d.d.f. F pr

Replicate 41.23 2 20.62 2.3 0.033

Genotype 273.81 24 11.09 14.9 <0.001

Season 54.90 2 27.45 136.9 <0.001

Genotype.season 71.80 48 1.50 136.9 0.037

**Dropping individual terms from full fixed model**

Fixed term Wald statistic n.d.f. F statistic d.d.f. F pr

Replicate 41.23 2 20.62 2.3 0.033

Genotype.season 71.80 48 1.50 136.9 0.037

**Standard errors of differences**

Replicate Season Genotype Genotype.season

Average 0.07275 0.07275 0.1888 0.3523

Maximum 0.2011 0.3638

Minimum 0.1810 0.3478

**viii. Response variate: Plant height (cm)**

Fixed model: Constant + replicate + genotype + season + genotype.season

Random model: Replicate.block

Number of units: 225

**Estimated variance components**

Random term component s.e.

Replicate.block 0.09 0.60

**Residual variance model**

Term Model(order) Parameter Estimate s.e.

Residual Identity Sigma2 13.78 1.67

**Deviance: -2*Log-Likelihood**

Deviance d.f.

626.91 146

**Tests for fixed effects**

Fixed term Wald statistic n.d.f. F statistic d.d.f. F pr

Replicate 17.86 2 8.93 6.1 0.016

Genotype 169.10 24 6.98 99.2 <0.001

Season 79.08 2 39.54 136.9 <0.001

Genotype.season 90.35 48 1.88 136.9 0.002

**Dropping individual terms from full fixed model**

Fixed term Wald statistic n.d.f. F statistic d.d.f. F pr

Replicate 17.86 2 8.93 6.1 0.016

Genotype.season 90.35 48 1.88 136.9 0.002

**Standard errors of differences**

Replicate Season Genotype Genotype.season

Average 0.6062 0.6062 1.763 3.038

Maximum 1.766 3.040

Minimum 1.755 3.031

**ix. Response variate: Kernels per spike**

Fixed model: Constant + replicate + genotype + season + genotype.season

Random model: Replicate.block

Number of units: 225

**Estimated variance components**

Random term component s.e.

Replicate.block 0.56 1.53

**Residual variance model**

Term Model(order) Parameter Estimate s.e.

Residual Identity Sigma2 31.48 3.81

**Deviance: -2*Log-Likelihood**

Deviance d.f.

750.39 146

**Tests for fixed effects**

Fixed term Wald statistic n.d.f. F statistic d.d.f. F pr

Replicate 19.35 2 9.67 6.4 0.012

Genotype 124.95 24 5.17 108.6 <0.001

Season 34.72 2 17.36 136.6 <0.001

Genotype.season 98.08 48 2.04 136.6 <0.001

**Dropping individual terms from full fixed model**

Fixed term Wald statistic n.d.f. F statistic d.d.f. F pr

Replicate 19.35 2 9.67 6.4 0.012

Genotype.season 98.08 48 2.04 136.6 <0.001

**Standard errors of differences**

Replicate Season Genotype Genotype.season

Average 0.9162 0.9162 2.695 4.609

Maximum 2.706 4.617

Minimum 2.665 4.581

**x. Response variate: Biomass (tha^-1^)**

Fixed model: Constant + replicate + genotype + season + genotype.season

Random model: Replicate.block

Number of units: 225

**Estimated variance components**

Random term component s.e.

Replicate.block 0.17 1.14

**Residual variance model**

Term Model(order) Parameter Estimate s.e.

Residual Identity Sigma2 24.80 3.01

**Deviance: -2*Log-Likelihood**

Deviance d.f.

713.90 146

**Tests for fixed effects**

Fixed term Wald statistic n.d.f. F statistic d.d.f. F pr

Replicate 2.38 2 1.19 5.4 0.372

Genotype 376.91 24 15.53 95.9 <0.001

Season 191.20 2 95.60 135.7 <0.001

Genotype.season 67.29 48 1.40 135.7 0.068

**Dropping individual terms from full fixed model**

Fixed term Wald statistic n.d.f. F statistic d.d.f. F pr

Replicate 2.38 2 1.19 5.4 0.372

Genotype.season 67.29 48 1.40 135.7 0.068

**Standard errors of differences**

Replicate Season Genotype Genotype.season

Average 0.8132 0.8132 2.366 4.076

Maximum 2.370 4.079

Minimum 2.355 4.066

**xi. Response variate: Grain yield (tha^-1^)**

Fixed model: Constant + replicate + genotype + season + genotype.season

Random model: Replicate.block

Number of units: 225

**Estimated variance components**

Random term component s.e.

Replicate.block -0.0055 0.0343

**Residual variance model**

Term Model(order) Parameter Estimate s.e.

Residual Identity Sigma2 0.848 0.1030

**Deviance: -2*Log-Likelihood**

Deviance d.f.

212.77 146

**Tests for fixed effects**

Fixed term Wald statistic n.d.f. F statistic d.d.f. F pr

Replicate 1.85 2 0.93 4.8 0.457

Genotype 755.56 24 31.01 79.3 <0.001

Season 237.84 2 118.92 135.7 <0.001

Genotype.season 133.82 48 2.79 135.7 <0.001

**Dropping individual terms from full fixed model**

Fixed term Wald statistic n.d.f. F statistic d.d.f. F pr

Replicate 1.85 2 0.93 4.8 0.457

Genotype.season 133.82 48 2.79 135.7 <0.001

**Standard errors of differences**

Replicate Season Genotype Genotype.season

Average 0.1504 0.1504 0.4304 0.7499

Maximum 0.4327 0.7520

Minimum 0.4296 0.7494

**xii. Response variate: Harvest index**

Fixed model: Constant + replicate + genotype + season + genotype.season

Random model: Replicate.block

Number of units: 225

**Estimated variance components**

Random term component s.e.

Replicate.block 0.000159 0.000153

**Residual variance model**

Term Model(order) Parameter Estimate s.e.

Residual Identity Sigma2 0.00199 0.000242

**Deviance: -2*Log-Likelihood**

Deviance d.f.

-675.55 146

**Tests for fixed effects**

Fixed term Wald statistic n.d.f. F statistic d.d.f. F pr

Replicate 0.35 2 0.18 7.4 0.842

Genotype 172.44 24 7.16 133.7 <0.001

Season 163.47 2 81.74 135.5 <0.001

Genotype.season 92.35 48 1.92 135.5 0.002

**Dropping individual terms from full fixed model**

Fixed term Wald statistic n.d.f. F statistic d.d.f. F pr

Replicate 0.35 2 0.18 7.4 0.842

Genotype.season 92.35 48 1.92 135.5 0.002

**Standard errors of differences**

Replicate Season Genotype Genotype.season

Average 0.007286 0.007286 0.02222 0.03711

Maximum 0.02252 0.03731

Minimum 0.02150 0.03643

**xiii. Response variate: 1000-kernel weight (g)**

Fixed model: Constant + replicate + genotype + season + genotype.season

Random model: Replicate.block

Number of units: 225

**Estimated variance components**

Random term component s.e.

Replicate.block 0.197 0.195

**Residual variance model**

Term Model(order) Parameter Estimate s.e.

Residual Identity Sigma2 3.021 0.364

**Deviance: -2*Log-Likelihood**

Deviance d.f.

407.51 146

**Tests for fixed effects**

Fixed term Wald statistic n.d.f. F statistic d.d.f. F pr

Replicate 0.41 2 0.20 8.5 0.819

Genotype 2327.35 24 96.67 132.4 <0.001

Season 121.75 2 60.87 137.5 <0.001

Genotype.season 350.87 48 7.31 137.5 <0.001

**Dropping individual terms from full fixed model**

Fixed term Wald statistic n.d.f. F statistic d.d.f. F pr

Replicate 0.41 2 0.20 8.5 0.819

Genotype.season 350.87 48 7.31 137.5 <0.001

**Standard errors of differences**

Replicate Season Genotype Genotype.season

Average 0.2838 0.2838 0.8605 1.443

Maximum 0.8705 1.449

Minimum 0.8354 1.419

**APPENDIX 2 Means of wheat genotypes evaluated for stem rust resistance over three cropping seasons**

| Genotype | GID | AUDPC | | | Final disease severity | | | Host plant response | | |
| --- | --- | --- | --- | --- | --- | --- | --- | --- | --- | --- |
|  |  | 22OS | 22MS | 23OS | 22OS | 22MS | 23OS | 22OS | 22MS | 23OS |
| G1 | 8789894 | 25 | 176 | 35 | 5 | 18 | 7 | RMR | MR | MR |
| G2 | 8790025 | 67 | 99 | 99 | 7 | 13 | 13 | M | MS | MS |
| G3 | 8790026 | 228 | 268 | 309 | 22 | 30 | 33 | MS | MS | MS |
| G4 | 8790027 | 6 | 39 | 22 | 1 | 7 | 5 | R | R | MR |
| G5 | 8790046 | 12 | 48 | 41 | 2 | 7 | 5 | M | RMR | M |
| G6 | 8790048 | 27 | 78 | 64 | 5 | 8 | 8 | M | M | M |
| G7 | 8790075 | 613 | 735 | 712 | 57 | 70 | 70 | S | S | S |
| G8 | 8790258 | 113 | 181 | 60 | 12 | 20 | 5 | M | MS | M |
| G9 | 8790275 | 36 | 118 | 99 | 5 | 15 | 12 | MR | MR | MR |
| G10 | 8790311 | 484 | 578 | 595 | 47 | 53 | 60 | MSS | MSS | MSS |
| G11 | 8790384 | 210 | 309 | 303 | 23 | 40 | 40 | MS | MSS | MSS |
| G12 | 8790668 | 21 | 158 | 111 | 4 | 22 | 12 | MR | MS | M |
| G13 | 8790751 | 32 | 55 | 44 | 4 | 8 | 5 | MR | RMR | M |
| G14 | 8790754 | 19 | 89 | 56 | 2 | 12 | 7 | MS | MS | M |
| G15 | 8790800 | 21 | 76 | 43 | 2 | 8 | 7 | RMR | RMR | M |
| G16 | 8790806 | 58 | 99 | 93 | 8 | 13 | 10 | MS | MR | M |
| G17 | 8790929 | 2 | 32 | 18 | 1 | 5 | 5 | R | RMR | R |
| G18 | 8790935 | 8 | 53 | 18 | 2 | 8 | 5 | R | RMR | RMR |
| G19 | 8790948 | 2 | 53 | 18 | 1 | 8 | 5 | R | RMR | RMR |
| G20 | 8790874 | 16 | 49 | 76 | 2 | 7 | 10 | M | RMR | M |
| G21 | 8790885 | 39 | 64 | 43 | 4 | 8 | 5 | MS | M | M |
| Checks | | | | | | | | | | |
| PBW 343 |  | 572 | 968 | 922 | 53 | 87 | 83 | MSS | S | S |
| K. Robin |  | 793 | 898 | 1015 | 70 | 87 | 90 | S | S | S |
| K.Kingbird |  | 99 | 147 | 93 | 10 | 22 | 12 | M | MS | M |
| K. Kasuku |  | 420 | 315 | 350 | 40 | 30 | 33 | MSS | MS | MSS |
| Mean |  | 157 | 227 | 210 | 15.5 | 24.3 | 21.9 |  |  |  |
| LSD_0.05_ |  | 0.57 | 0.31 | 0.33 | 10.94 | 11.46 | 7.71 |  |  |  |

AUDPC - Area under disease progress curve, 22OS - 2022 off-season, 22MS - 2022 main-season, 23OS - 2023 off-season, GID – genotype identification number, G – genotype

**APPENDIX 3** Means of agronomic traits for 25 wheat lines evaluated for stem rust resistance for 3 cropping seasons at KALRO, Njoro.

|  | Days to heading | | | |  | Days to flowering | | | |  | | Days to physiological maturity(days) | | | |
| --- | --- | --- | --- | --- | --- | --- | --- | --- | --- | --- | --- | --- | --- | --- | --- |
| Genotype | 22OS | 22MS | 23OS | Overall  Mean | | 22OS | 22MS | 23OS | Overall  mean | | 22OS | | 22MS | 23OS | Overall  mean |
| G1 | 70 | 69 | 69 | 69 |  | 73 | 75 | 74 | 74 |  | | 113 | 124 | 130 | 122 |
| G2 | 68 | 68 | 69 | 68 |  | 73 | 76 | 74 | 74 |  | | 113 | 120 | 130 | 121 |
| G3 | 68 | 66 | 67 | 67 |  | 74 | 76 | 74 | 74 |  | | 112 | 125 | 123 | 120 |
| G4 | 65 | 66 | 67 | 66 |  | 71 | 75 | 74 | 73 |  | | 116 | 124 | 126 | 122 |
| G5 | 74 | 72 | 71 | 72 |  | 77 | 79 | 77 | 77 |  | | 120 | 125 | 130 | 125 |
| G6 | 66 | 65 | 68 | 66 |  | 71 | 73 | 73 | 72 |  | | 115 | 124 | 128 | 122 |
| G7 | 70 | 71 | 70 | 70 |  | 73 | 77 | 75 | 75 |  | | 114 | 126 | 128 | 123 |
| G8 | 66 | 66 | 69 | 67 |  | 72 | 75 | 74 | 74 |  | | 112 | 121 | 127 | 120 |
| G9 | 61 | 65 | 68 | 65 |  | 68 | 72 | 74 | 71 |  | | 114 | 125 | 130 | 123 |
| G10 | 67 | 67 | 70 | 68 |  | 72 | 75 | 75 | 74 |  | | 111 | 121 | 123 | 119 |
| G11 | 73 | 75 | 74 | 74 |  | 77 | 82 | 81 | 80 |  | | 114 | 127 | 129 | 123 |
| G12 | 83 | 82 | 81 | 82 |  | 88 | 89 | 88 | 88 |  | | 129 | 128 | 137 | 131 |
| G13 | 68 | 69 | 69 | 69 |  | 74 | 76 | 74 | 75 |  | | 119 | 122 | 126 | 122 |
| G14 | 69 | 69 | 70 | 69 |  | 74 | 76 | 76 | 75 |  | | 119 | 123 | 130 | 124 |
| G15 | 71 | 71 | 69 | 70 |  | 75 | 77 | 74 | 75 |  | | 118 | 125 | 125 | 123 |
| G16 | 72 | 70 | 70 | 71 |  | 75 | 77 | 76 | 76 |  | | 123 | 125 | 130 | 126 |
| G17 | 65 | 66 | 67 | 66 |  | 72 | 75 | 74 | 73 |  | | 113 | 123 | 132 | 122 |
| G18 | 68 | 67 | 67 | 67 |  | 72 | 75 | 74 | 74 |  | | 116 | 121 | 132 | 123 |
| G19 | 67 | 65 | 69 | 67 |  | 71 | 75 | 75 | 74 |  | | 118 | 121 | 133 | 124 |
| G20 | 71 | 69 | 69 | 70 |  | 75 | 77 | 75 | 76 |  | | 119 | 125 | 127 | 124 |
| G21 | 74 | 71 | 69 | 71 |  | 78 | 78 | 76 | 78 |  | | 121 | 127 | 129 | 125 |
| G22 | 68 | 69 | 72 | 70 |  | 74 | 76 | 76 | 75 |  | | 122 | 124 | 120 | 122 |
| G23 | 70 | 69 | 69 | 70 |  | 73 | 76 | 75 | 75 |  | | 111 | 121 | 119 | 117 |
| G24 | 64 | 64 | 66 | 65 |  | 69 | 71 | 73 | 71 |  | | 109 | 118 | 120 | 116 |
| G25 | 56 | 64 | 67 | 62 |  | 61 | 71 | 73 | 68 |  | | 111 | 124 | 125 | 120 |

Appendix 3 continued

|  | Grain filling period (days) | | | |  | Spike length (cm) | | | |  | Plant height (cm) | | | |
| --- | --- | --- | --- | --- | --- | --- | --- | --- | --- | --- | --- | --- | --- | --- |
| Genotype | 22OS | 22MS | 23OS | Overall mean |  | 22OS | 22MS | 23OS | Overall mean | | 22OS | 22MS | 23OS | Overall mean |
| G1 | 40 | 49 | 55 | 48 |  | 9 | 9 | 9 | 9 |  | 93 | 90 | 88 | 90 |
| G2 | 40 | 45 | 55 | 47 |  | 10 | 9 | 9 | 9 |  | 89 | 95 | 96 | 93 |
| G3 | 38 | 49 | 50 | 46 |  | 9 | 9 | 9 | 9 |  | 86 | 91 | 95 | 91 |
| G4 | 45 | 49 | 52 | 49 |  | 9 | 8 | 9 | 9 |  | 91 | 93 | 92 | 92 |
| G5 | 43 | 47 | 53 | 48 |  | 9 | 9 | 10 | 9 |  | 89 | 89 | 98 | 92 |
| G6 | 44 | 51 | 55 | 50 |  | 9 | 8 | 9 | 9 |  | 86 | 87 | 94 | 89 |
| G7 | 40 | 49 | 53 | 48 |  | 9 | 9 | 9 | 9 |  | 93 | 96 | 98 | 96 |
| G8 | 40 | 46 | 54 | 47 |  | 11 | 10 | 10 | 10 |  | 97 | 98 | 102 | 99 |
| G9 | 47 | 53 | 57 | 52 |  | 10 | 9 | 9 | 9 |  | 92 | 96 | 100 | 96 |
| G10 | 39 | 46 | 48 | 45 |  | 9 | 9 | 9 | 9 |  | 91 | 92 | 95 | 93 |
| G11 | 37 | 45 | 48 | 43 |  | 9 | 9 | 8 | 9 |  | 94 | 97 | 98 | 96 |
| G12 | 41 | 39 | 49 | 43 |  | 10 | 9 | 10 | 10 |  | 102 | 96 | 106 | 102 |
| G13 | 45 | 46 | 52 | 47 |  | 9 | 9 | 9 | 9 |  | 90 | 93 | 95 | 92 |
| G14 | 45 | 47 | 54 | 49 |  | 10 | 9 | 10 | 10 |  | 92 | 92 | 98 | 94 |
| G15 | 43 | 48 | 51 | 47 |  | 9 | 9 | 9 | 9 |  | 91 | 89 | 98 | 93 |
| G16 | 48 | 48 | 54 | 50 |  | 10 | 9 | 10 | 9 |  | 91 | 87 | 99 | 92 |
| G17 | 41 | 48 | 58 | 49 |  | 10 | 9 | 10 | 10 |  | 92 | 96 | 97 | 95 |
| G18 | 44 | 46 | 59 | 49 |  | 10 | 10 | 10 | 10 |  | 86 | 96 | 92 | 92 |
| G19 | 47 | 46 | 58 | 50 |  | 9 | 9 | 10 | 9 |  | 88 | 94 | 95 | 92 |
| G20 | 44 | 48 | 52 | 48 |  | 9 | 8 | 9 | 9 |  | 88 | 88 | 98 | 91 |
| G21 | 43 | 48 | 52 | 48 |  | 10 | 9 | 10 | 9 |  | 92 | 84 | 93 | 90 |
| G22 | 48 | 48 | 44 | 47 |  | 9 | 9 | 9 | 9 |  | 88 | 90 | 97 | 92 |
| G23 | 37 | 45 | 44 | 42 |  | 10 | 9 | 9 | 9 |  | 98 | 91 | 94 | 95 |
| G24 | 41 | 47 | 47 | 45 |  | 8 | 8 | 8 | 8 |  | 83 | 90 | 88 | 87 |
| G25 | 50 | 53 | 52 | 51 |  | 10 | 9 | 10 | 10 |  | 84 | 89 | 89 | 87 |
|  |  |  |  |  |  |  |  |  |  |  |  |  |  |  |

Appendix 3 continued

|  | Kernels per spike | | | |  | Biomass (t/ha) | | | |  | Grain yield (t/ha) | | | |
| --- | --- | --- | --- | --- | --- | --- | --- | --- | --- | --- | --- | --- | --- | --- |
| Genotype | 22OS | 22MS | 23OS | Overall mean | | 22OS | 22MS | 23OS | Overall mean | | 22OS | 22MS | 23OS | Overall mean |
| G1 | 45.5 | 53.6 | 45.5 | 48.2 |  | 27.14 | 29.94 | 41.31 | 32.80 |  | 7.72 | 3.60 | 7.79 | 6.37 |
| G2 | 56.3 | 50.3 | 56.0 | 54.2 |  | 18.33 | 28.75 | 35.54 | 27.54 |  | 5.01 | 4.17 | 7.33 | 5.50 |
| G3 | 57.3 | 42.7 | 52.9 | 50.9 |  | 16.13 | 19.29 | 27.68 | 21.03 |  | 3.37 | 2.65 | 4.58 | 3.53 |
| G4 | 49.4 | 56.7 | 55.5 | 53.9 |  | 18.04 | 27.98 | 28.33 | 24.78 |  | 5.64 | 4.28 | 5.85 | 5.26 |
| G5 | 58.7 | 51.2 | 55.7 | 55.2 |  | 20.83 | 20.65 | 29.40 | 23.63 |  | 5.26 | 2.99 | 4.37 | 4.21 |
| G6 | 54.5 | 40.6 | 46.0 | 47.0 |  | 17.50 | 19.29 | 23.33 | 20.04 |  | 3.57 | 2.39 | 3.90 | 3.29 |
| G7 | 53.5 | 58.5 | 47.9 | 53.3 |  | 15.95 | 27.02 | 24.35 | 22.44 |  | 1.62 | 2.07 | 2.43 | 2.04 |
| G8 | 58.1 | 52.7 | 55.1 | 55.3 |  | 26.43 | 26.79 | 40.71 | 31.31 |  | 7.23 | 4.41 | 8.45 | 6.70 |
| G9 | 52.8 | 51.4 | 46.9 | 50.4 |  | 24.35 | 25.95 | 36.13 | 28.81 |  | 5.38 | 2.77 | 7.25 | 5.14 |
| G10 | 52.5 | 50.3 | 45.0 | 49.3 |  | 19.88 | 25.18 | 25.83 | 23.63 |  | 3.65 | 3.59 | 3.27 | 3.50 |
| G11 | 61.1 | 62.6 | 55.6 | 59.8 |  | 24.70 | 29.82 | 29.23 | 27.92 |  | 5.28 | 3.11 | 5.06 | 4.48 |
| G12 | 55.5 | 51.3 | 47.9 | 51.6 |  | 24.88 | 34.06 | 42.02 | 33.65 |  | 4.17 | 3.20 | 4.32 | 3.90 |
| G13 | 52.8 | 42.4 | 49.3 | 48.2 |  | 20.12 | 27.23 | 29.52 | 25.63 |  | 5.60 | 3.39 | 6.36 | 5.11 |
| G14 | 51.3 | 44.7 | 48.0 | 48.0 |  | 21.90 | 19.88 | 35.18 | 25.65 |  | 5.55 | 2.90 | 6.03 | 4.82 |
| G15 | 51.3 | 42.8 | 45.5 | 46.5 |  | 18.27 | 22.92 | 28.33 | 23.17 |  | 4.04 | 3.11 | 5.96 | 4.37 |
| G16 | 60.5 | 45.3 | 50.9 | 52.2 |  | 18.33 | 17.98 | 28.75 | 21.69 |  | 4.49 | 2.97 | 4.90 | 4.12 |
| G17 | 58.3 | 53.9 | 49.0 | 53.8 |  | 28.93 | 30.36 | 36.34 | 31.88 |  | 7.19 | 4.25 | 9.24 | 6.89 |
| G18 | 55.5 | 58.7 | 53.1 | 55.8 |  | 23.33 | 34.11 | 43.33 | 33.59 |  | 6.65 | 4.65 | 8.52 | 6.61 |
| G19 | 59.1 | 49.7 | 47.9 | 52.2 |  | 27.92 | 29.88 | 51.13 | 36.31 |  | 8.12 | 4.09 | 9.21 | 7.14 |
| G20 | 51.3 | 41.4 | 40.9 | 44.5 |  | 13.33 | 18.27 | 27.08 | 19.56 |  | 3.74 | 2.97 | 4.42 | 3.71 |
| G21 | 58.1 | 43.1 | 50.0 | 50.4 |  | 15.06 | 14.94 | 25.30 | 18.43 |  | 3.24 | 1.88 | 3.97 | 3.03 |
| G22 | 30.5 | 37.7 | 47.1 | 38.4 |  | 8.99 | 16.96 | 17.14 | 14.37 |  | 0.22 | 1.03 | 1.29 | 0.85 |
| G23 | 54.5 | 41.3 | 44.9 | 46.9 |  | 5.06 | 10.54 | 10.06 | 8.55 |  | 0.27 | 0.81 | 1.67 | 0.92 |
| G24 | 55.0 | 42.3 | 53.9 | 50.4 |  | 13.99 | 19.52 | 21.01 | 18.17 |  | 3.55 | 2.67 | 4.38 | 3.53 |
| G25 | 50.5 | 51.9 | 49.7 | 50.7 |  | 14.05 | 18.27 | 25.71 | 19.35 |  | 2.38 | 2.70 | 3.43 | 2.84 |

Appendix 2 continued

|  | Harvest index | | | |  | Thousand kernel weight (g) | | | |
| --- | --- | --- | --- | --- | --- | --- | --- | --- | --- |
| Genotype | 22OS | 22MS | 23OS | Overall mean | | 22OS | 22MS | 23OS | Overall mean |
| G1 | 0.28 | 0.12 | 0.19 | 0.20 |  | 36.17 | 35.39 | 36.22 | 35.93 |
| G2 | 0.27 | 0.15 | 0.21 | 0.21 |  | 29.47 | 33.93 | 29.25 | 30.88 |
| G3 | 0.21 | 0.14 | 0.17 | 0.17 |  | 26.02 | 29.97 | 23.92 | 26.63 |
| G4 | 0.31 | 0.15 | 0.22 | 0.23 |  | 33.34 | 33.54 | 29.93 | 32.27 |
| G5 | 0.26 | 0.15 | 0.15 | 0.19 |  | 31.70 | 31.89 | 26.77 | 30.12 |
| G6 | 0.20 | 0.12 | 0.17 | 0.17 |  | 30.06 | 29.31 | 24.89 | 28.09 |
| G7 | 0.10 | 0.08 | 0.10 | 0.09 |  | 18.57 | 24.98 | 18.98 | 20.84 |
| G8 | 0.27 | 0.16 | 0.21 | 0.22 |  | 38.75 | 39.71 | 42.15 | 40.20 |
| G9 | 0.23 | 0.12 | 0.20 | 0.18 |  | 34.03 | 33.75 | 35.18 | 34.32 |
| G10 | 0.18 | 0.14 | 0.13 | 0.15 |  | 22.87 | 33.03 | 20.91 | 25.60 |
| G11 | 0.22 | 0.11 | 0.17 | 0.17 |  | 27.44 | 33.87 | 25.86 | 29.06 |
| G12 | 0.18 | 0.10 | 0.10 | 0.13 |  | 26.50 | 32.52 | 31.91 | 30.31 |
| G13 | 0.28 | 0.13 | 0.22 | 0.21 |  | 33.40 | 35.74 | 28.39 | 32.51 |
| G14 | 0.27 | 0.15 | 0.17 | 0.19 |  | 34.85 | 34.62 | 30.85 | 33.44 |
| G15 | 0.23 | 0.14 | 0.21 | 0.19 |  | 33.47 | 32.71 | 30.67 | 32.28 |
| G16 | 0.25 | 0.17 | 0.17 | 0.19 |  | 27.15 | 29.52 | 24.33 | 27.00 |
| G17 | 0.25 | 0.14 | 0.28 | 0.22 |  | 36.33 | 36.30 | 41.44 | 38.03 |
| G18 | 0.29 | 0.14 | 0.20 | 0.21 |  | 30.57 | 32.62 | 37.32 | 33.51 |
| G19 | 0.30 | 0.14 | 0.19 | 0.21 |  | 31.14 | 31.54 | 37.53 | 33.40 |
| G20 | 0.28 | 0.17 | 0.16 | 0.20 |  | 29.04 | 31.22 | 25.67 | 28.64 |
| G21 | 0.22 | 0.13 | 0.16 | 0.17 |  | 27.81 | 28.57 | 23.12 | 26.50 |
| G22 | 0.03 | 0.06 | 0.07 | 0.05 |  | 11.20 | 17.75 | 16.47 | 15.14 |
| G23 | 0.06 | 0.08 | 0.20 | 0.11 |  | 15.52 | 17.90 | 11.71 | 15.04 |
| G24 | 0.26 | 0.14 | 0.21 | 0.20 |  | 30.12 | 28.97 | 28.64 | 29.24 |
| G25 | 0.17 | 0.15 | 0.13 | 0.15 |  | 26.92 | 32.02 | 25.20 | 28.05 |
